# An individualized prognostic nomogram integrating clinical and pathological features in pediatric IgA vasculitis nephritis

**DOI:** 10.3389/fmed.2026.1771283

**Published:** 2026-03-12

**Authors:** Yueheng Gan, Li Xiao, Shaojun Li, Anshuo Wang, Sijie Yu, Xuejun Yang, Mo Wang, Qiu Li, Han Chan, Haiping Yang

**Affiliations:** 1 Department of Nephrology, Children’s Hospital of Chongqing Medical University, National Clinical Research Center for Children and Adolescents’ Health and Diseases, Ministry of Education Key Laboratory of Child Development and Disorders, Chongqing Key Laboratory of Pediatric Metabolism and Inflammatory Diseases, Chongqing, China; 2 Big Data Center for Children’s Medical Care, Children’s Hospital of Chongqing Medical University, Chongqing, China; 3 Department of Emergency, Children’s Hospital of Chongqing Medical University, Chongqing, China

**Keywords:** children, cox regression, estimated glomerular filtration rate, IgA vasculitis nephritis, prognostic model

## Abstract

**Objective:**

To develop and validate an individualized prognostic nomogram integrating clinical and pathological features for estimating the risk of renal function decline in children with IgA vasculitis nephritis (IgAVN).

**Methods:**

In this single-center retrospective cohort, 603 children with biopsy-confirmed IgAVN and ≥12 months of follow-up were included. The primary endpoint was a composite of eGFR <90 mL/min/1.73 m^2^ or a ≥ 30% decline from baseline, first occurring after 12 months of follow-up. The cohort was randomly split into training (80%) and validation (20%) sets. Variable selection was performed using elastic-net regression with five-fold cross-validation, followed by backward stepwise Cox proportional hazards modelling.

**Results:**

Over a median follow-up of 48.7 months (IQR: 28.5–73.3 months), 68 patients (11.3%) reached the endpoint. The final model identified five independent predictors: male sex (HR = 2.12), renal IgA deposit 3+ (HR = 2.66), ISKDC grade IV–VI (HR = 4.63), Oxford T1/2 lesions (HR = 4.72), and baseline serum creatinine. The model showed strong and consistent discrimination in the validation set (*C*-index = 0.796; 60 month AUC = 0.859; 84 month AUC = 0.850), adequate calibration, and provided positive net clinical benefit on decision curve analysis. A practical nomogram was developed for risk estimation. Kaplan-Meier analysis demonstrated significantly lower event-free survival in the high-risk group (log-rank *p* < 0.001).

**Conclusion:**

We successfully developed and validated an individualized prognostic nomogram. This tool integrates key clinical and pathological features to quantify the risk of renal function decline in children with IgAVN shortly after renal biopsy, providing a basis for personalized management decisions.

## 1 Introduction

IgA vasculitis (IgAV), formerly known as Henoch-Schönlein purpura, is a systemic small-vessel vasculitis characterized by the deposition of IgA-containing immune complexes. Renal involvement, referred to as IgAVN, is the major determinant of long-term prognosis (1). The annual incidence of IgAVN in children has been estimated at 15–70 per million, and it is one of the most common secondary glomerular diseases in childhood. Although most children present with mild renal abnormalities and experience favorable outcomes, long-term studies indicate that approximately 1–36% progress to chronic kidney disease (CKD) (2), and up to 20–25% may develop end-stage renal disease (ESRD) within 10–20 years (3, 4). This risk of renal deterioration imposes a substantial burden on patients, families, and healthcare systems. Consequently, early identification of children at high risk for unfavorable renal outcomes is crucial to guide personalized management strategies.

Several baseline factors have been associated with adverse renal prognosis in IgAVN, including nephrotic range proteinuria, impaired renal function at diagnosis, hypertension, and specific histopathological features (5–8). However, many existing studies are based on univariate analyses or traditional multivariable models that do not fully integrate clinical, laboratory, and pathological data. Furthermore, some reported models rely on biomarkers not routinely available in clinical practice (9, 10), while others employ short-term or cross-sectional outcomes that do not adequately account for follow-up duration or censoring (11). These limitations compromise the predictive accuracy and clinical utility of existing prognostic tools.

Advances in machine learning have created new opportunities to improve risk prediction for complex renal diseases. Methods such as elastic net regression can efficiently handle high-dimensional data, identify stable predictors, and reduce the influence of multicollinearity (12–14). When combined with time-to-event analytical approaches, these techniques allow more effective use of longitudinal follow-up data and may enhance the prediction of medium- and long-term renal outcomes.

In this context, we aimed to develop and validate a robust, clinically actionable tool to address this gap. By leveraging a large single-center retrospective cohort with comprehensive data, our objective was to construct an individualized prognostic nomogram that integrates key clinical and pathological features. This tool is designed to quantify the personalized risk of estimate glomerular filtration rate (eGFR) decline at 5 and 7 years in children with IgAVN shortly after renal biopsy, thereby providing a practical basis for tailored management decisions.

## 2 Materials and methods

### 2.1 Study design and participants

This single-center retrospective cohort study included children diagnosed with IgAVN at the Department of Nephrology, Children’s Hospital of Chongqing Medical University between December 2012 and October 2024. The diagnosis of IgAVN was established in accordance with the 2025 International Pediatric Nephrology Association (IPNA) clinical practice recommendations (15). Specifically, inclusion criteria were: (1) diagnosis of IgAV based on the presence of palpable purpura (predominantly on the lower limbs and buttocks) without thrombocytopenia or coagulopathy, as per the EULAR/PRINTO/PRES Ankara 2008 classification criteria (16); (2) evidence of kidney involvement, defined as the presence of hematuria and/or proteinuria (15); (3) renal biopsy performed to confirm glomerulonephritis characterized by dominant or co-dominant glomerular IgA deposition on immunofluorescence; (4) age at renal biopsy <17 years; (5) availability of complete baseline clinical, laboratory, and histopathological data; and (6) regular follow-up for at least 12 months.

Exclusion criteria comprised: (1) follow-up duration <12 months or incomplete follow-up data; (2) a renal biopsy specimen containing fewer than 10 glomeruli for adequate histopathological evaluation; (3) concomitant diseases that may affect renal prognosis, such as hepatitis B virus infection, lupus nephritis, or Alport syndrome.

All renal biopsies were performed after obtaining informed consent from the parents or legal guardians. The study was approved by the Institutional Ethics Committee of our hospital (Approval no. 2025-417), and the requirement for informed consent for participation was waived due to the retrospective nature of the study.

### 2.2 Data collection and variable processing

Demographic, clinical, laboratory, pathological, treatment, and follow-up data were collected from the electronic medical record system for the period between December 2012 and October 2025.

Demographic variables included age, sex, height, weight, and mean arterial pressure (MAP). Clinical data included the time from disease onset to renal biopsy, recurrence of purpura, presence of gross hematuria, treatment before and after biopsy, and follow-up duration. Laboratory parameters included urinary red blood cell count (URBC), 24 h urinary protein (24-UP), neutrophil-to-lymphocyte ratio (NLR), total bile acid (TBA), blood urea (UREA), serum creatinine (SCr), serum uric acid (UA), cystatin C (CysC), serum immunoglobulin A, M, G, and E (IgA, IgM, IgG, IgE), complement C3, complement C4, and the IgA/C3 ratio. Pathological data included the International Study of Kidney Diseases in Children (ISKDC) classification (17) and the Oxford classification (MEST-C score) (18), as well as the intensity of immunofluorescence staining for IgA, IgM, IgG, C3, and C1q.

Renal biopsies were independently evaluated by two pathologists blinded to clinical data. IgA deposition intensity was graded semi-quantitatively as 1+, 2+, or 3+ on immunofluorescence microscopy. Tubular atrophy and interstitial fibrosis were scored according to the Oxford classification as T0 (0–25%), T1 (26–50%), or T2 (>50%). Disagreements were resolved by consensus, and all reports were reviewed by a senior nephrologist trained in renal pathology.

Cases with more than 10% missing data fields were excluded from analysis. Variables with ≥10% missingness were removed. For variables with <10% missingness, missing values were imputed using the mean for normally distributed data and the median for non-normally distributed data. A comparison of variable distributions before and after imputation is provided in Supplementary Table 1. No statistically significant differences were observed (all *p* > 0.05), confirming that the imputation process did not introduce systematic bias into the dataset.

### 2.3 Outcome definition

To avoid including transient renal impairment, the primary endpoint was assessed using a 12 month landmark approach: only patients who first met the eGFR <90 mL/min/1.73 m^2^ or ≥30% decline criteria at any time after the 12 month follow-up visit were considered to have reached the endpoint. Events occurring within the first 12 months after biopsy were not counted. For patients with baseline eGFR <90 mL/min/1.73 m^2^ at biopsy, endpoint status was determined based on renal function after the 12 month landmark: those with normalized eGFR (≥90 mL/min/1.73 m^2^) at last follow-up (≥12 months) were classified as non-events; those with persistent eGFR <90 mL/min/1.73 m^2^ beyond 12 months were classified as events.

The eGFR was calculated using the full-age-spectrum FAS equation (19). The follow-up period was measured from the date of renal biopsy until the occurrence of the endpoint event, loss to follow-up, or the study end date (31 October 2025), whichever occurred first.

### 2.4 Statistical analysis

Statistical analyses were performed using R version 4.4.3 and SPSS 25.0. Continuous variables with a normal distribution were expressed as mean ± standard deviation and compared using the *t*-test. Variables not normally distributed were expressed as median (interquartile range) and compared using the Mann-Whitney *U* tests. Categorical variables were presented as frequencies and percentages, and compared using the Chi-square test or Fisher’s exact test.

To develop the prediction model, the dataset was randomly split into a training set and a test set in an 8:2 ratio using stratified sampling based on the outcome to ensure similar event rates between the two sets. In the training set, an elastic-net regression with five-fold cross-validation (*α* = 0.5) was used to select candidate predictors. Variables that were selected in at least four of the five folds were identified as consensus predictors to ensure robustness. These consensus variables were then incorporated into a backward stepwise Cox proportional hazards model with variable retention guided by the Akaike Information Criterion (AIC) to construct the final multivariable prediction model. The proportional hazards assumption was tested using Schoenfeld residuals. For continuous predictors retained in the final model, potential nonlinearity was examined using restricted cubic splines with three knots; model fit was compared using the likelihood ratio test and AIC.

Model discrimination was evaluated using Harrell’s *C*-index and time-dependent receiver operating characteristic (ROC) curve analysis (AUC). Calibration was assessed using calibration plots and the Brier score. Clinical utility was assessed by decision curve analysis (DCA). All time-dependent performance measures (AUC, Brier score, calibration, and DCA) were calculated using methods that incorporate inverse probability of censoring weighting (IPCW) based on Kaplan-Meier (KM) estimation of the censoring distribution. Bootstrap resampling (1,000 iterations) was used to calculate 95% confidence intervals (CI) for all performance metrics to assess model stability. A nomogram was developed based on the final model. Risk stratification was performed using KM curves, and group differences were compared with the log-rank test. All statistical tests were two-sided, and a *p* value <0.05 was considered statistically significant.

## 3 Results

### 3.1 Baseline clinical and pathological characteristics

A total of 603 patients were included in the final analysis. The median age at disease onset was 10.00 (7.92, 12.32) years, and 343 patients (56.88%) were male. The median follow-up duration after renal biopsy was 48.67 (28.45, 73.28) months. During follow-up, 68 children (11.27%) experienced the primary endpoint of eGFR decline.

Regarding clinical presentation, the majority of patients presented with either the hematuria and proteinuria type (208 cases, 34.49%) or the nephrotic syndrome type (280 cases, 46.43%). At the time of renal biopsy, 90 children (14.93%) already had impaired renal function (eGFR <90 mL/min/1.73m^2^). Based on the 12 month landmark definition, among the 90 children with baseline eGFR <90 mL/min/1.73 m^2^ at biopsy, 65 (72.2%) had normalized eGFR (≥90 mL/min/1.73 m^2^) at their last follow-up (≥12 months) and were classified into the non-event group, as their renal impairment proved reversible. The remaining 25 (27.8%) had persistent eGFR <90 mL/min/1.73 m^2^ beyond 12 months and were classified into the event group, reflecting sustained renal dysfunction consistent with CKD.

During the entire follow-up period, the median eGFR slope was 1.07 (−2.59, 5.46) mL/min/1.73 m^2^/year in the non-event group, significantly higher than that in the event group [−4.86 (−9.12, 0.58) mL/min/1.73 m^2^/year]. Compared with the non-event group, children in the event group had significantly lower baseline eGFR, higher SCr and CysC levels, and a higher proportion of ISKDC grades IV–VI and Oxford classification T1/T2 (all *p* < 0.05).

In terms of post-biopsy treatment, no significant differences were observed between the two groups, except that plasma exchange was used more frequently in the event group (*p* < 0.05). In addition, the intensity of immunofluorescence staining in renal tissue did not differ significantly between the groups in univariate analysis. Detailed comparisons of baseline characteristics are presented in Table 1.

**Table 1:** Comparison of baseline characteristics between the two groups.

Variables	Total (*n* = 603)	Non-event group (*n* = 535)	Event group (*n* = 68)	Statistic	*p*
Age, month, *M* (Q_1_, Q_3_)	10.00 (7.92, 12.32)	9.87 (7.75, 12.21)	11.53 (8.82, 13.00)	*Z* = −2.84	0.004
Gender, *n* (%)				*χ*^2^ = 2.70	0.100
Female	260 (43.12)	237 (44.30)	23 (33.82)		
Male	343 (56.88)	298 (55.70)	45 (66.18)		
Height, cm, *M* (Q_1_, Q_3_)	138.00 (126.50, 153.00)	137.00 (126.00, 153.00)	140.00 (131.50, 156.00)	*Z* = −2.06	0.040
Weight, kg, M (Q_1_, Q_3_)	32.50 (25.00, 43.00)	32.00 (25.00, 42.50)	38.50 (28.00, 44.62)	*Z* = −2.46	0.014
MAP, mmHg, *M* (Q_1_, Q_3_)	80.67 (74.33, 87.67)	80.33 (74.33, 87.00)	82.00 (77.00, 91.08)	*Z* = −1.79	0.074
Time from diagnosis to renal biopsy, month, *M* (Q_1_, Q_3_)	0.20 (0.13, 0.52)	0.20 (0.10, 0.47)	0.27 (0.13, 2.11)	*Z* = −2.22	0.027
Follow-up time, month, *M* (Q_1_, Q_3_)	48.67 (28.45, 73.28)	47.77 (27.38, 73.68)	53.08 (36.44, 67.86)	*Z* = −0.90	0.368
Gross hematuria, *n* (%)				*χ*^2^ = 3.74	0.053
No	464 (76.95)	418 (78.13)	46 (67.65)		
Yes	139 (23.05)	117 (21.87)	22 (32.35)		
Recurrence of purpuric rash, *n* (%)				*χ*^2^ = 0.82	0.366
No	376 (62.35)	337 (62.99)	39 (57.35)		
Yes	227 (37.65)	198 (37.01)	29 (42.65)		
Clinical classification, *n* (%)				—	0.003*
Isolated hematuria	23 (3.81)	22 (4.11)	1 (1.47)		
Isolated proteinuria	28 (4.64)	25 (4.67)	3 (4.41)		
Hematuria and proteinuria	208 (34.49)	186 (34.77)	22 (32.35)		
Acute nephritic syndrome	35 (5.80)	30 (5.61)	5 (7.35)		
Nephrotic syndrome	280 (46.43)	254 (47.48)	26 (38.24)		
Rapidly progressive glomerulonephritis	7 (1.16)	3 (0.56)	4 (5.88)		
Chronic glomerulonephritis	22 (3.65)	15 (2.80)	7 (10.29)		
First urine protein neg time, *n* (%)				*χ*^2^ = 23.43	<0.001
≤1 month	91 (15.09)	81 (15.14)	10 (14.71)		
1–6 month	355 (58.87)	325 (60.75)	30 (44.12)		
6–12 month	69 (11.44)	64 (11.96)	5 (7.35)		
>12 month	64 (10.61)	47 (8.79)	17 (25.00)		
No remission	24 (3.98)	18 (3.36)	6 (8.82)		
Laboratory test results					
eGFR, ml/min/1.73 m^2^, *M* (Q_1_, Q_3_)	111.70 (97.06, 126.02)	112.69 (99.32, 126.02)	97.40 (74.86, 126.33)	*Z* = −3.12	0.002
24 h-UP, mg/kg, *M* (Q_1_, Q_3_)	61.20 (23.74, 103.72)	60.57 (23.38, 100.78)	76.80 (24.50, 119.20)	*Z* = −1.21	0.225
URBC, /μL, *M* (Q_1_, Q_3_)	121.00 (27.00, 450.00)	118.00 (27.00, 459.00)	152.00 (32.75, 400.00)	*Z* = −0.42	0.675
NLR, *M* (Q_1_, Q_3_)	2.43 (1.43, 4.66)	2.43 (1.43, 4.64)	2.80 (1.48, 5.45)	*Z* = −0.95	0.340
TBA, mmol/L, *M* (Q_1_, Q_3_)	2.70 (1.50, 5.00)	2.70 (1.50, 5.00)	2.60 (1.50, 4.43)	*Z* = −0.62	0.532
UREA, mmol/L, *M* (Q_1_, Q_3_)	4.60 (3.50, 6.00)	4.53 (3.50, 5.90)	4.86 (3.68, 6.54)	*Z* = −1.54	0.123
SCr, μmol/L, *M* (Q_1_, Q_3_)	43.00 (36.00, 52.00)	43.00 (36.00, 51.00)	52.00 (36.75, 68.50)	*Z* = −3.22	0.001
UA, μmol/L, *M* (Q_1_, Q_3_)	277.00 (229.00, 344.60)	274.00 (231.00, 337.50)	310.50 (204.25, 368.12)	*Z* = −0.71	0.477
CysC, mg/L, *M* (Q_1_, Q_3_)	0.95 (0.81, 1.10)	0.95 (0.81, 1.08)	1.08 (0.90, 1.35)	*Z* = −3.74	<0.001
IgG, g/L, *M* (Q_1_, Q_3_)	7.70 (5.67, 9.98)	7.71 (5.67, 10.00)	7.70 (5.63, 9.82)	*Z* = −0.18	0.860
IgA, g/L, *M* (Q_1_, Q_3_)	2.20 (1.63, 2.92)	2.21 (1.62, 2.98)	2.08 (1.68, 2.75)	*Z* = −1.21	0.228
IgM, g/L, *M* (Q_1_, Q_3_)	1.23 (0.94, 1.58)	1.23 (0.94, 1.58)	1.23 (0.90, 1.42)	*Z* = −1.20	0.232
IgE, IU/mL, *M* (Q_1_, Q_3_)	32.00 (12.55, 76.70)	32.00 (11.85, 81.10)	32.00 (15.17, 62.92)	*Z* = −0.10	0.918
C3, g/L, *M* (Q_1_, Q_3_)	0.92 (0.79, 1.06)	0.92 (0.78, 1.05)	0.95 (0.83, 1.14)	*Z* = −1.66	0.097
C4, g/L, *M* (Q_1_, Q_3_)	0.19 (0.15, 0.24)	0.19 (0.15, 0.24)	0.21 (0.16, 0.24)	*Z* = −1.15	0.252
IgA/C3, *M* (Q_1_, Q_3_)	2.39 (1.76, 3.29)	2.41 (1.76, 3.32)	2.30 (1.72, 3.06)	*Z* = −1.46	0.144
eGFR slope, ml/min/1.73 m^2^/year, *M* (Q_1_, Q_3_)	0.51 (−3.53, 4.82)	1.07 (−2.59, 5.46)	−4.86 (−9.12, 0.58)	*Z* = −5.70	<0.001
Treatment					
RASB, *n* (%)				*χ*^2^ = 0.00	1.000
No	16 (2.65)	14 (2.62)	2 (2.94)		
Yes	587 (97.35)	521 (97.38)	66 (97.06)		
GC, *n* (%)				*χ*^2^ = 0.00	0.950
No	23 (3.81)	21 (3.93)	2 (2.94)		
Yes	580 (96.19)	514 (96.07)	66 (97.06)		
Plasma exchange, *n* (%)				*χ*^2^ = 5.72	0.017
No	593 (98.34)	529 (98.88)	64 (94.12)		
Yes	10 (1.66)	6 (1.12)	4 (5.88)		
Renal pathological					
ISKDC classification, *n* (%)				*χ*^2^ = 17.33	<0.001
I + II + III	577 (95.69)	519 (97.01)	58 (85.29)		
IV + V + VI	26 (4.31)	16 (2.99)	10 (14.71)		
Oxford classification					
M, *n* (%)				*χ*^2^ = 0.87	0.351
0	307 (50.91)	276 (51.59)	31 (45.59)		
1	296 (49.09)	259 (48.41)	37 (54.41)		
E, *n* (%)				*χ*^2^ = 0.11	0.741
0	505 (83.75)	449 (83.93)	56 (82.35)		
1	98 (16.25)	86 (16.07)	12 (17.65)		
S, *n* (%)				*χ*^2^ = 1.74	0.187
0	480 (79.60)	430 (80.37)	50 (73.53)		
1	123 (20.40)	105 (19.63)	18 (26.47)		
T, *n* (%)				—	<0.001
0	595 (98.67)	533 (99.63)	62 (91.18)		
1/2	8 (1.33)	2 (0.37)	6 (8.82)		
C, *n* (%)				*χ*^2^ = 8.54	0.014
0	254 (42.12)	230 (42.99)	24 (35.29)		
1	267 (44.28)	240 (44.86)	27 (39.71)		
2	82 (13.60)	65 (12.15)	17 (25.00)		
Immunofluorescence					
IgA deposit, *n* (%)				*χ*^2^ = 1.31	0.519
1+	88 (14.59)	81 (15.14)	7 (10.29)		
2+	103 (17.08)	92 (17.20)	11 (16.18)		
3+	412 (68.33)	362 (67.66)	50 (73.53)		
IgM deposit, *n* (%)				*χ*^2^ = 0.75	0.387
−	211 (34.99)	184 (34.39)	27 (39.71)		
+	392 (65.01)	351 (65.61)	41 (60.29)		
C3 deposit, *n* (%)				*χ*^2^ = 2.71	0.439
−	175 (29.02)	156 (29.16)	19 (27.94)		
+	428 (70.98)	379 (70.84)	49 (72.06)		
IgG deposit, *n* (%)				*χ*^2^ = 0.27	0.601
−	518 (85.90)	461 (86.17)	57 (83.82)		
+	85 (14.10)	74 (13.83)	11 (16.18)		
C1q deposit, *n* (%)				—	0.569
−	596 (98.84)	529 (98.88)	67 (98.53)		
+	7 (1.16)	6 (1.12)	1 (1.47)		

*Z*, Mann-Whitney test; *χ*^2^, Chi-square test; —, Fisher exact; *, Simulated *p*-value; *M*, Median; Q_1_, 1st quartile; Q_3_, 3st quartile; MAP, mean arterial pressure; eGFR, estimate glomerular filtration rate; 24-UP: 24 h urinary protein; URBC, urinary red blood cells; NLR, neutrophil to lymphocyte ratio; TBA, total bile acid; UREA, blood urea; SCr, serum creatinine; UA, serum uric acid; CysC, cystatin C; IgG, immunoglobulin G; IgA, immunoglobulin A; IgM, immunoglobulin M; IgE, immunoglobulin E; C3, complement 3; C4, complement 4; IgA/C3, IgA to C3 ratio; RASB, renin angiotensin system blockade; GC, glucocorticoid; ISKDC, international study of kidney diseases.

### 3.2 Variable selection and model development

**Figure 1:**
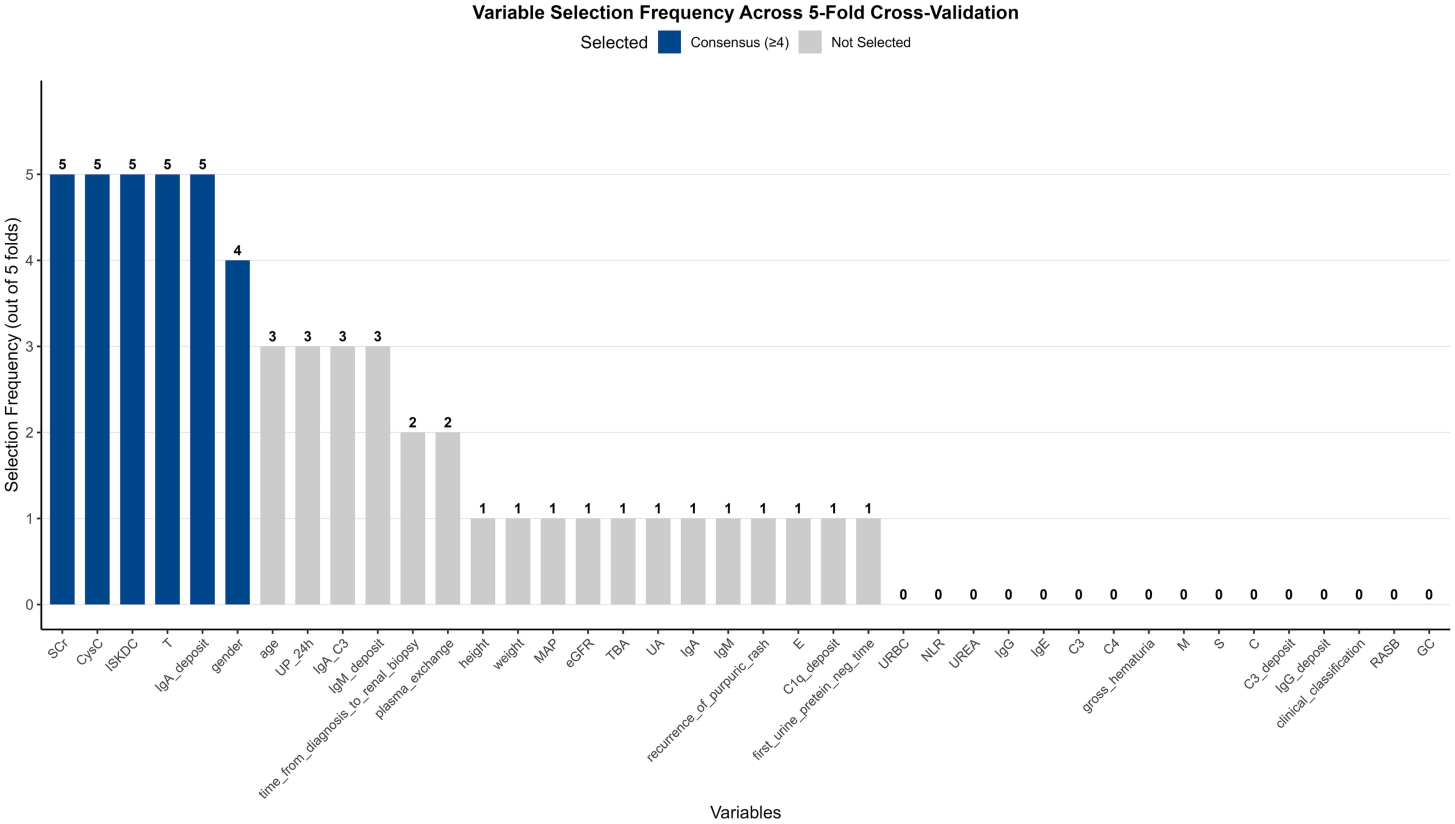
Variable selection frequency from the elastic-net regression with five-fold cross-validation.

After stratified random sampling in an 8:2 ratio, the training set included 483 children (53 events) and the test set included 120 children (15 events). Baseline characteristics were balanced between the two sets (Supplementary Table 2). In the training set, elastic-net regression with five-fold cross-validation identified 6 consensus variables from 40 candidate predictors: sex, renal IgA deposition intensity, ISKDC grade, Oxford classification T, baseline SCr, and CysC (Figure 1). The mean performance of these consensus variables across the five folds was: *C*-index = 0.689 ± 0.091, 60 month AUC = 0.678 ± 0.142, and 80 month AUC = 0.708 ± 0.083. Detailed performance metrics for each fold are presented in Supplementary Table 3.

**Figure 2:**
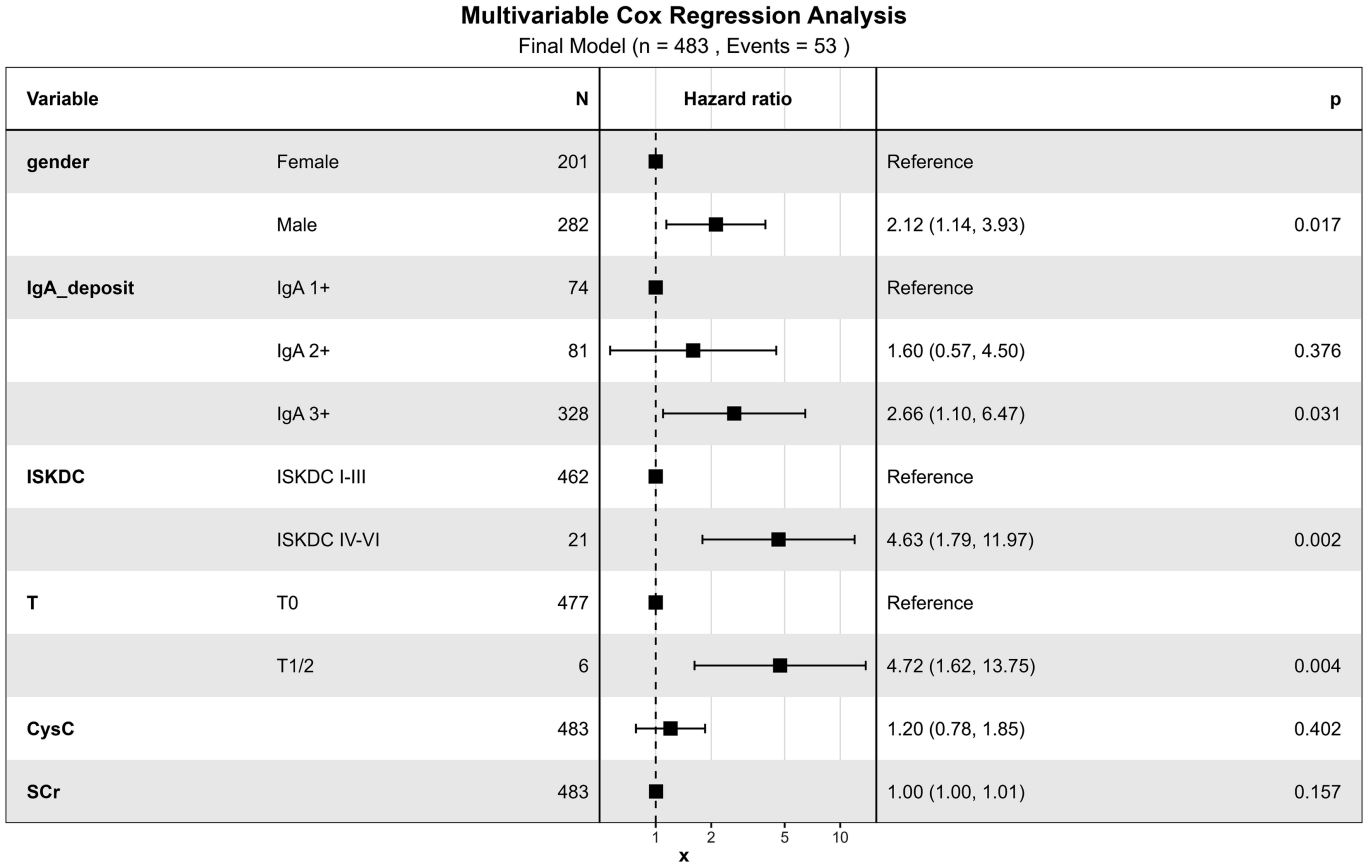
Multivariate Cox regression forest plot.

Using the consensus variables, we performed backward stepwise Cox regression based on the AIC in the training set. The final model retained five predictors: sex, renal IgA deposition intensity, ISKDC grade, Oxford classification T lesions, and baseline SCr. In the multivariable Cox analysis (Figure 2), male sex [hazard ratio (HR) = 2.12, 95% CI: 1.14–3.93, *p* = 0.017], renal IgA deposition intensity of 3+ (HR = 2.66, 95% CI: 1.10–6.47, *p* = 0.031), ISKDC grade IV–VI (HR = 4.63, 95% CI: 1.79–11.97, *p* = 0.002), and Oxford classification T1/2 (HR = 4.72, 95% CI: 1.62–13.75, *p* = 0.004) were all independent predictors of poor renal outcomes. Baseline SCr was also retained in the final model based on the AIC criterion, with an HR of 1.01 for each 1 μmol/L increase (95% CI: 1.00–1.01, *p* = 0.157).

To further evaluate the contribution of SCr to the prognostic model, we performed a sensitivity analysis by refitting the model without this variable (Supplementary Table 4). Removal of SCr resulted in a modest reduction in predictive performance: the *C*-index decreased from 0.796 to 0.756 in the test set (Δ −0.040), the 60 month AUC decreased from 0.859 to 0.795 (Δ −0.064), and the 84 month AUC decreased from 0.850 to 0.771 (Δ − 0.079). These findings suggest that although SCr as a continuous variable yields an HR close to 1, it nonetheless contributes meaningful prognostic information that enhances the overall predictive accuracy of the model.

### 3.3 Model performance evaluation

The proportional hazards assumption was satisfied for all predictors in the final model, as indicated by the global Schoenfeld test (*χ*^2^ = 1.59, d*f* = 6, *p* = 0.95) and individual variable tests (all *p* > 0.05; Supplementary Table 5). The corresponding residual plots are provided in Supplementary Figure 1.

To assess the functional form of SCr—the only continuous predictor in the final model—we compared a linear specification with a restricted cubic spline (3 knots). The likelihood ratio test showed no significant improvement with the spline term (*χ*^2^ = 0.32, d*f* = 1, *p* = 0.57), and the AIC was slightly lower for the linear model (528.86 vs. 530.54). The fitted spline effect is displayed in Supplementary Figure 2. These results support the linear representation of SCr in the model.

### 3.4 Model performance evaluation

In the training set, bootstrap internal validation (1,000 iterations) yielded the following performance metrics: *C*-index = 0.730 (95% CI: 0.649–0.802), 60 month AUC = 0.735 (95% CI: 0.629–0.827), 84 month AUC = 0.756 (95% CI: 0.657–0.846), and a Brier score of 0.114 (95% CI: 0.087–0.140). In the independent test set (*n* = 120), model performance was as follows: *C*-index = 0.796 (95% CI: 0.684–0.899), 60 month AUC = 0.859 (95% CI: 0.750–0.956), 84 month AUC = 0.850 (95% CI: 0.692–0.973), and a Brier score of 0.097 (95% CI: 0.048–0.146). Detailed performance metrics are shown in Table 2. Time-dependent ROC curves at 60 and 84 months for both the training and test sets are presented in Supplementary Figure 3.

**Table 2:** The performance of the model in the training set and the test set (after bootstrap validation).

Dataset	*C*-index	60 month AUC	84 month AUC	Brier score
Train	0.730 (95% CI: 0.649–0.802)	0.735 (95% CI: 0.629–0.827)	0.756 (95% CI: 0.657–0.846)	0.114 (95% CI: 0.087–0.140)
Test	0.796 (95% CI: 0.684–0.899)	0.859 (95% CI: 0.750–0.956)	0.850 (95% CI: 0.692–0.973)	0.097 (95% CI: 0.048–0.146)

**Figure 3:**
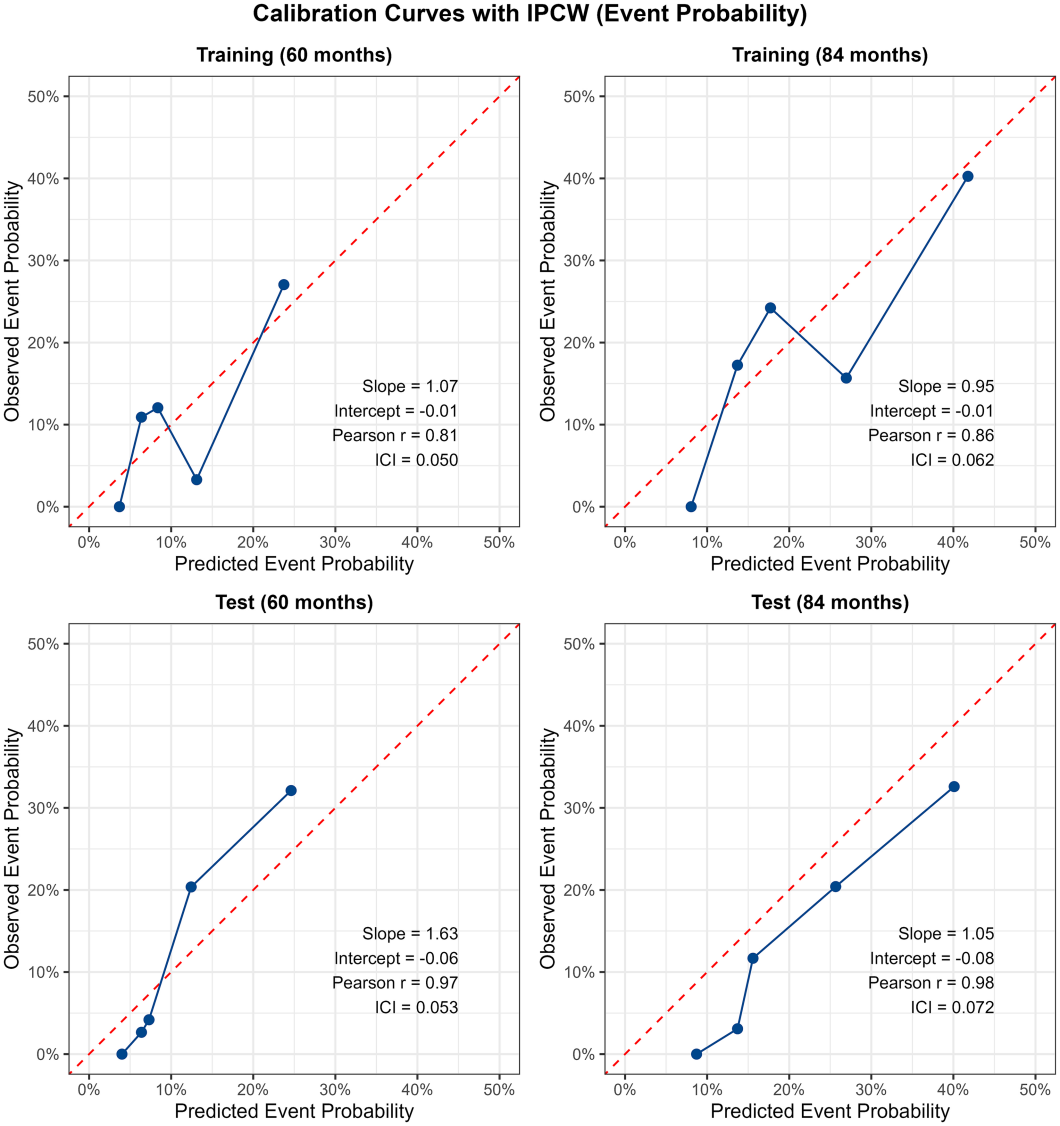
Model calibration curves (training set and test set, 60 months and 84 months).

Calibration curves for the training and test sets at 60 and 84 months are shown in Figure 3. In the training set, the calibration slopes were 1.07 (60 months) and 0.95 (84 months), intercepts −0.01 and −0.01, Pearson correlation coefficients 0.81 and 0.86, and integrated calibration index (ICI) values 0.050 and 0.062. In the test set, slopes ranged from 1.05 to 1.63, intercepts from −0.08 to −0.06, Pearson *r* from 0.97 to 0.98, and ICI from 0.053 to 0.072. The slightly higher slope of 1.63 at 60 months in the test set indicates a modest underestimation of risk in the highest risk stratum, but overall the calibration remained acceptable given the low event rate (11.3%) and the small ICI values (all <0.08). These results confirm that the model’s predicted event probabilities align well with observed outcomes.

**Figure 4:**
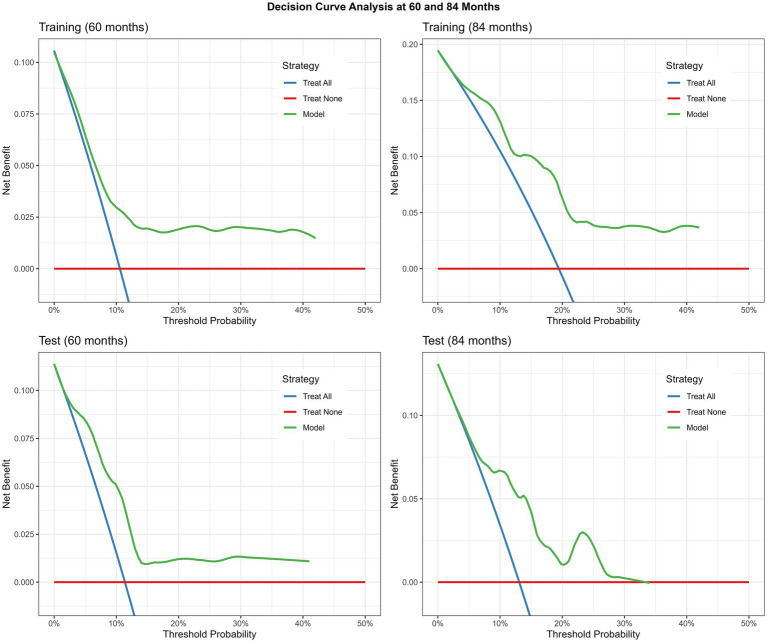
Model decision curve analysis.

Decision curve analysis at 60 and 84 months (Figure 4) showed that the application of the prediction model provided greater net benefit than either the “treat-all” or “treat-none” strategies across the range of threshold probabilities, supporting its adequate appropriate clinical utility.

In the full cohort (*n* = 603), the number of patients remaining at risk at 60 and 84 months was 232 and 92, respectively, with corresponding cumulative censoring rates of 54.7 and 75.5% (Supplementary Table 6). Of note, the 84 month estimates should be interpreted with caution due to the increasing censoring rate at this time point.

### 3.5 Incremental value of additional predictors

We compared the final model with three simpler baseline models fitted on the training set: an ISKDC-only model, an ISKDC + Oxford T model, and a clinical-only model (gender and SCr). Their predictive performance on the test set is summarized in Supplementary Table 7. The final model consistently outperformed all three simpler models across all metrics, with substantially higher *C*-index and time-dependent AUC values, while the baseline models showed considerably lower discriminative ability. Decision curve analysis (Supplementary Figure 4) further demonstrated the clinical utility of the final model, with net benefit exceeding that of the baseline models at a 10% threshold probability in both training and test sets at 60 and 84 months (Supplementary Table 8). These findings confirm that integrating IgA intensity and multiple clinical-pathological features enhances both predictive accuracy and clinical utility.

### 3.6 Nomogram and risk stratification

**Figure 5:**
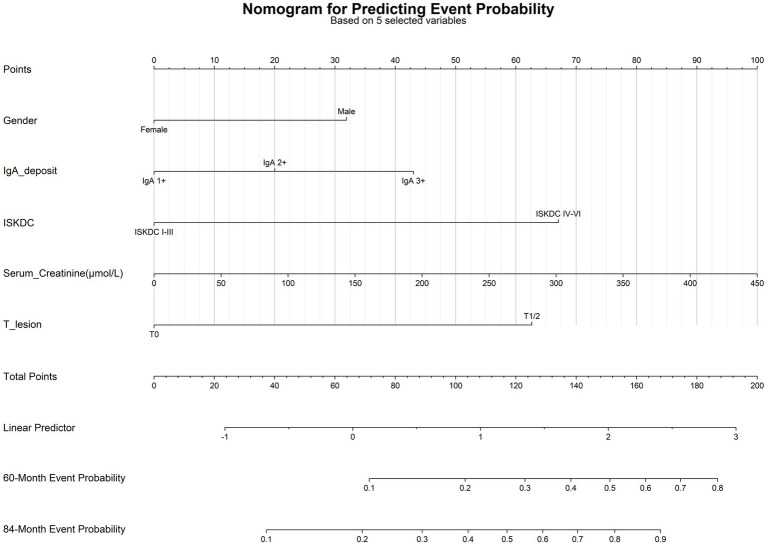
Nomogram for predicting the 60/84 months risk of renal function decline.

A nomogram (Figure 5) was constructed based on the final Cox model to provide a practical tool for clinical risk estimation. By summing the points assigned to each predictor, clinicians can estimate an individual patient’s probability of experiencing the endpoint event at 60 and 84 months. For example, a male patient (32 points) with IgA deposition graded as 3+ on renal biopsy (43 points), ISKDC grade II (0 points), a baseline SCr of 100 μmol/L at the time of biopsy (22.5 points), and an Oxford classification T1 (22.5 points) would have a total score of approximately 160 points. This corresponds to an estimated event probability of about 57% at 60 months and 85% at 84 months.

**Figure 6:**
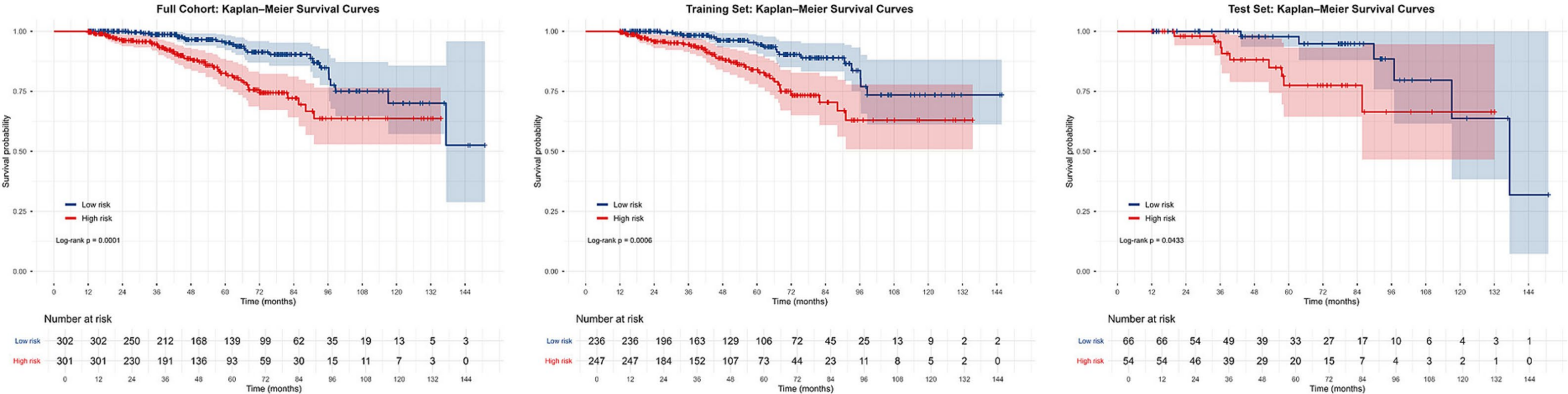
Kaplan-Meier survival curves based on model risk scores (full cohort, training set, test set).

To facilitate risk stratification, patients in the full cohort, training set, and test set were dichotomized into high- and low-risk groups based on the median model-derived risk score. KM survival analysis (Figure 6) showed that event-free survival was significantly lower in the high-risk group than in the low-risk group across all datasets (log-rank *p* < 0.001). KM analyses stratified by the five key predictors were also performed (Supplementary Figure 5). For example, children with Oxford classification T0 lesions had significantly higher event-free survival at 84 months compared with those with T1/T2 (log-rank *p* < 0.001).

## 4 Discussion

In this large, single-center cohort study, we developed and validated an individualized prognostic nomogram for estimating the 5 year and 7 year risk of renal function decline in children with IgAVN. The nomogram synthesizes five readily available clinical and pathological variables—sex, renal IgA deposit, ISKDC grade, Oxford classification T lesions, and baseline SCr—into a single, interpretable risk score. The model demonstrated favorable discrimination, calibration, and clinical utility upon rigorous evaluation in both training and independent test sets.

Our approach and the resulting tool offer several translational advantages over previous prognostic studies. First, the use of elastic net regression for initial variable selection helped address common problems such as overfitting and multicollinearity in traditional approaches, thereby improving model stability and generalizability. Second, the application of the Cox proportional hazards model allowed appropriate handling of censored follow up data and enabled estimation of the probability of renal function decline at specific time points, providing more timely and dynamic risk information. Third, model performance was rigorously evaluated through bootstrap internal validation and assessment in an independent test set, ensuring a comprehensive and reliable validation. The most significant advance, however, lies in the implementation of the model as a clinical nomogram. This provides a user-friendly, visual interface that translates complex statistical predictions into an immediate, quantitative risk estimate at the bedside, facilitating personalized clinical decision-making soon after renal biopsy.

Notably, our model identified male sex as an independent risk factor for poor renal outcome in IgAVN (HR = 2.12), a finding consistent with several previous observational studies (7, 11, 20, 21). A meta-analysis by Chan et al. (22) and other reports also indicated that boys are more likely to develop severe renal involvement (23). The underlying mechanism may involve the immuno-regulatory effects of sex hormones. Androgens may promote Th17 cell differentiation and suppress Treg activity, which could amplify immune-mediated inflammation (24). In contrast, estrogens may enhance Treg function and exert protective effects on the kidney (25, 26). However, not all studies have reported significant sex-related differences in outcome (27, 28); these discrepancies may be attributed to variations in sample size, ethnic background, or follow-up duration. In our large cohort, multivariable analysis confirmed male sex as a significant predictor, suggesting that boys with IgAVN may warrant closer clinical monitoring.

We also observed that children with an IgA deposition intensity graded as 3+ faced a higher risk of renal function decline (HR = 2.66). Previous studies have similarly suggested that the intensity of IgA deposition may influence IgAVN prognosis (29). This finding reflects the central disease mechanism of IgAVN, in which immune complexes formed by galactose-deficient IgA1 and corresponding autoantibodies deposit in the mesangium and trigger inflammatory injury. A higher deposit may indicate a greater immune-complex burden and more pronounced mesangial activation. Although immunofluorescence scoring is routinely reported in renal biopsy pathology, most prognostic models have not incorporated it as a quantitative predictor. Our study identified it as a stable predictive factor, highlighting that the extent of immune complex deposition itself provides important prognostic information beyond standard pathological classifications.

ISKDC grading is a classic system for assessing the severity of acute lesions in IgAVN. In this study, children with grades IV–VI, which often include crescent formation and or sclerosis, had a markedly higher risk compared with those with grades I–III (HR = 4.63). This finding aligns with results from most studies worldwide (27, 30). Crescents, particularly cellular crescents, indicate severe active proliferative injury and are hallmarks of rapid renal function decline. Our results further confirm the central importance of ISKDC grading in predicting outcomes in pediatric IgAVN.

Tubular atrophy and interstitial fibrosis in the Oxford classification (T1/2) represent irreversible chronic kidney damage. In this study, T1 or T2 lesions were strong independent predictors of poor outcome (HR = 4.72), which aligns with findings from adult IgA nephropathy and an increasing number of pediatric IgAVN studies (1, 8, 31–33). These chronic lesions typically result from persistent inflammation, ischemia, or protein-related toxicity, and they disrupt normal renal microstructure and impair the potential for functional recovery. It is noteworthy that while the ISKDC grading primarily reflects acute active injury, the T lesions capture chronic fibrotic damage. The inclusion of both ISKDC grade and T lesions in the final model underscores that a comprehensive renal assessment in children with IgAVN should account for both the severity of current inflammatory activity and the extent of accumulated chronic injury. These two dimensions offer complementary and essential prognostic information.

Baseline SCr, a fundamental clinical indicator of renal function, was retained in the final model based on its established clinical relevance and contribution to overall model performance. Although it was not statistically significant in the multivariable analysis (*p* = 0.157), the AIC-based selection strategy, which aims to optimize overall predictive performance rather than rely on a single *p*-value (34), supported its retention. This decision was further validated by our sensitivity analysis, which demonstrated that removal of SCr from the model led to a modest but consistent reduction in predictive accuracy (*C*-index decreased by 0.040 in the test set). The interpretation of SCr’s near-unity hazard ratio (1.01 per 1 μmol/L increase) requires consideration of both statistical and clinical perspectives. Statistically, as a continuous variable, each 1 μmol/L increment in SCr corresponds to only a 1% increase in risk. However, clinically relevant changes in SCr typically involve increments of 10–30 μmol/L or more, translating to 10–35% increases in risk, which is meaningful in clinical decision-making. Moreover, SCr may capture renal function information that is partially overlapping with but not fully redundant to the pathological variables (ISKDC grade and Oxford T lesions), explaining its modest but consistent contribution to model performance (35, 36). In clinical practice, a higher baseline SCr indicates pre-existing renal impairment at diagnosis, providing essential information on renal status that complements the biopsy-based pathological variables included in the model.

Furthermore, while heavy proteinuria is a well-recognized risk factor for progression to ESRD (30, 37, 38), it was not retained in our final model after multivariable adjustment. This may reflect that proteinuria’s prognostic information is largely captured by the pathological variables (particularly ISKDC grade and Oxford T lesions) and baseline renal function (SCr) included in the model. Notably, in our cohort, 192 children (31.84%) still had varying degrees of proteinuria at their last follow-up, including 157 in the non-event group. This observation suggests that persistent proteinuria, even in the absence of other high-risk features, may warrant extended follow-up to monitor for potential late-onset disease progression. Future studies incorporating serial proteinuria measurements could further clarify its dynamic role in IgAVN prognosis.

Several predictive models have been developed in IgAV and IgAVN. Bi et al. (39) constructed a diagnostic nomogram to predict renal involvement in children with IgAV. In contrast, our study focuses on prognostic prediction in patients with established IgAVN, using Cox regression to account for time-to-event dynamics. Our model integrates clinical and pathological features to provide individualized risk estimates for long-term renal function decline.

Several limitations should be considered. First, as a single-center retrospective analysis, selection bias cannot be excluded. Second, given the considerable heterogeneity in treatment strategies, detailed therapeutic regimens and their adjustments over time were not systematically incorporated into the model. This omission may have led to an underestimation of the impact of treatment response on long-term outcomes. Third, while our model demonstrated robust performance at 84 months, the censoring rate at this time point reached 75.5%, which warrants cautious interpretation. We have provided detailed risk tables in the Supplementary materials to facilitate transparency, and readers should consider this when applying the 84 month predictions in clinical practice. Future multicenter prospective studies and external validation are needed to confirm our findings and enhance model applicability across diverse populations.

In conclusion, we have developed and validated an individualized prognostic nomogram that integrates key clinical and pathological features to provide a quantified, time-specific risk estimate for children with IgAVN shortly after renal biopsy. This tool moves beyond qualitative assessment by offering a user-friendly interface for bedside application. To translate this innovation into clinical practice, future work should focus on external validation across diverse populations and, ultimately, on evaluating its impact in guiding risk-based management protocols.

## Data Availability

The raw data supporting the conclusions of this article will be made available by the authors, without undue reservation.
