# Supplementary material for: An individualized prognostic nomogram integrating clinical and pathological features in pediatric IgA vasculitis nephritis
Source: Front Med (Lausanne). 2026 Mar 12;13:1771283. doi: 10.3389/fmed.2026.1771283 (PMC13017366; doi:10.3389/fmed.2026.1771283)

**Supplementary Table 1. Comparison of variable distributions before and after imputation**

| variable | % Missing | Before Imputation | After Imputation | P |
| --- | --- | --- | --- | --- |
| TBA, mmol/L, M (Q₁, Q₃) | 3.3% | 2.70 (1.50, 5.10) | 2.70 (1.50, 5.00) | 0.987 |
| CysC, mg/L, M (Q₁, Q₃) | 2.1% | 0.95 (0.81, 1.10) | 0.95 (0.81, 1.10) | 0.995 |
| IgG, g/L, M (Q₁, Q₃) | 1.3% | 7.70 (5.60, 10.00) | 7.70 (5.67, 9.98) | 0.996 |
| IgA, g/L, M (Q₁, Q₃) | 1.1% | 2.20 (1.61, 2.93) | 2.20 (1.63, 2.92) | 1.000 |
| IgM, g/L, M (Q₁, Q₃) | 1.1% | 1.23 (0.92, 1.58) | 1.23 (0.94, 1.58) | 0.996 |
| IgE, g/L, M (Q₁, Q₃) | 1.3% | 32.00 (12.40, 79.30) | 32.00 (12.55, 76.70) | 0.961 |
| C3, g/L, M (Q₁, Q₃) | 1.3% | 0.92 (0.79, 1.06) | 0.92 (0.79, 1.06) | 0.957 |
| C4, g/L, M (Q₁, Q₃) | 1.3% | 0.19 (0.15, 0.24) | 0.19 (0.15, 0.24) | 0.953 |
| IgA/C3, M (Q₁, Q₃) | 1.3% | 2.40 (1.74, 3.30) | 2.39 (1.76, 3.29) | 0.937 |

**Supplementary Table 2. The baseline characteristics of the training set and test set**

| Variables | Train (n = 483) | Test (n = 120) | *P* |
| --- | --- | --- | --- |
|  |  |  |  |
| Age, month, M (Q₁, Q₃) | 9.92(7.92,12.25) | 10.25(7.92,12.43) | 0.486 |
| Gender, n(%) |  |  | 0.164 |
| Female | 282 (58.4%) | 61 (50.8%) |  |
| Male | 201 (41.6%) | 59 (49.2%) |  |
| Height, cm, M (Q₁, Q₃) | 137.00(126.00,153.00) | 140.00(127.00,154.00) | 0.649 |
| Weight, Kg, M (Q₁, Q₃) | 32.50(25.00,42.75) | 32.00(25.00,44.00) | 0.587 |
| MAP, mmHg, M (Q₁, Q₃) | 80.67(74.50,87.67) | 80.33(74.33,87.75) | 0.649 |
| Time From Diagnosis To Renal Biopsy, month, M (Q₁, Q₃) | 0.20(0.10,0.48) | 0.20(0.13,0.58) | 0.709 |
| Follow-up time, month, M (Q₁, Q₃) | 47.1(27.2,71.6) | 55.0(34.4,79.9) | 0.368 |
| Gross Hematuria, n(%) |  |  | 0.048 |
| No | 363 (75.2%) | 101 (84.2%) |  |
| Yes | 120 (24.8%) | 19 (15.8%) |  |
| Recurrence Of Purpuric Rash, n(%) |  |  | 0.262 |
| No | 307 (63.6%) | 69 (57.5%) |  |
| Yes | 176 (36.4%) | 51 (42.5%) |  |
| Clinical Classification, n(%) |  |  | NA |
| Isolated hematuria | 19 (3.9%) | 4 (3.3%) |  |
| Isolated proteinuria | 23 (4.8%) | 5 (4.2%) |  |
| Hematuria and proteinuria | 171 (35.4%) | 37 (30.8%) |  |
| Acute nephritic syndrome | 22 (4.6%) | 13 (10.8%) |  |
| Nephrotic syndrome | 221 (45.8%) | 59 (49.2%) |  |
| Rapidly progressive glomerulonephritis | 6 (1.2%) | 1 (0.8%) |  |
| Chronic glomerulonephritis | 21 (4.3%) | 1 (0.8%) |  |
| First Urine Protein Neg Time, n(%) |  |  | 0.196 |
| ≤1 month | 73 (15.1%) | 18 (15.0%) |  |
| 1-6 month | 285 (59.0%) | 70 (58.3%) |  |
| 6-12 month | 55 (11.4%) | 14 (11.7%) |  |
| ＞12 month | 55 (11.4%) | 9 (7.5%) |  |
| No remission | 15 (3.1%) | 9 (7.5%) |  |
| **Laboratory test results** |  |  |  |
| eGFR, ml/min/1.73m^2^, M (Q₁, Q₃) |  |  |  |
| 24h-UP, mg/kg, M (Q₁, Q₃) | 60.62(24.06,107.82) | 64.67(21.59,97.00) | 0.953 |
| URBC, /μL, M (Q₁, Q₃) | 114.00(26.00,493.00) | 145.00(32.00,413.25) | 0.800 |
| NLR, M (Q₁, Q₃) | 2.48(1.42,4.77) | 2.27(1.64,3.71) | 0.602 |
| TBA, mmol/L, M (Q₁, Q₃) | 2.70(1.50,4.85) | 2.85(1.50,5.32) | 0.690 |
| UREA, mmol/L, M (Q₁, Q₃) | 4.60(3.56,6.05) | 4.50(3.28,5.60) | 0.153 |
| SCr, μmol/L, M (Q₁, Q₃) | 43.00(36.00,52.00) | 43.00(37.45,54.00) | 0.674 |
| UA, μmol/L, M (Q₁, Q₃) | 278.00(229.00,339.00) | 274.50(228.00,360.25) | 0.728 |
| CysC, mg/L, M (Q₁, Q₃) | 0.95(0.81,1.10) | 0.96(0.84,1.09) | 0.507 |
| IgG, g/L, M (Q₁, Q₃) | 7.70(5.58,10.00) | 7.82(5.87,9.86) | 0.573 |
| IgA, g/L, M (Q₁, Q₃) | 2.20(1.61,2.92) | 2.20(1.78,3.07) | 0.522 |
| IgM, g/L, M (Q₁, Q₃) | 1.24(0.92,1.56) | 1.23(1.01,1.61) | 0.523 |
| IgE, IU/mL, M (Q₁, Q₃) | 32.00(13.20,78.35) | 31.45(10.88,69.25) | 0.485 |
| C3, g/L, M (Q₁, Q₃) | 0.92(0.78,1.06) | 0.95(0.80,1.05) | 0.282 |
| C4, g/L, M (Q₁, Q₃) | 0.19(0.15,0.23) | 0.20(0.17,0.25) | 0.018 |
| IgA/C3, M (Q₁, Q₃) | 2.41(1.74,3.27) | 2.30(1.78,3.35) | 0.983 |
| eGFR Slope, ml/min/1.73m^2^/year, M (Q₁, Q₃) | 0.73(-3.56,5.10) | 0.25(-3.41,2.66) | 0.423 |
| **Treatment** |  |  |  |
| RASB, n(%) |  |  | 1.000 |
| No | 13 (2.7%) | 3 (2.5%) |  |
| Yes | 470 (97.3%) | 117 (97.5%) |  |
| GC, n(%) |  |  | 0.306 |
| No | 16 (3.3%) | 7 (5.8%) |  |
| Yes | 467 (96.7%) | 113 (94.2%) |  |
| Plasma Exchange, n(%) |  |  | 1.000 |
| No | 475 (98.3%) | 118 (98.3%) |  |
| Yes | 8 (1.7%) | 2 (1.7%) |  |
| **Renal pathological** |  |  |  |
| ISKDC classification, n(%) |  |  | 1.000 |
| I＋II＋III | 462 (95.7%) | 115 (95.8%) |  |
| IV＋V＋VI | 21 (4.3%) | 5 (4.2%) |  |
| Oxford classification |  |  |  |
| M, n(%) |  |  | 0.121 |
| 0 | 254 (52.6%) | 53 (44.2%) |  |
| 1 | 229 (47.4%) | 67 (55.8%) |  |
| E, n(%) |  |  | 0.783 |
| 0 | 406 (84.1%) | 99 (82.5%) |  |
| 1 | 77 (15.9%) | 21 (17.5%) |  |
| S, n(%) |  |  | 0.130 |
| 0 | 378 (78.3%) | 102 (85.0%) |  |
| 1 | 105 (21.7%) | 18 (15.0%) |  |
| T, n(%) |  |  | 0.662 |
| 0 | 477 (98.8%) | 118 (98.3%) |  |
| 1/2 | 6 (1.2%) | 2 (1.7%) |  |
| C, n(%) |  |  | 0.725 |
| 0 | 206 (42.7%) | 48 (40.0%) |  |
| 1 | 210 (43.5%) | 57 (47.5%) |  |
| 2 | 67 (13.9%) | 15 (12.5%) |  |
| Immunofluorescence |  |  |  |
| IgA deposit, n(%) |  |  | 0.583 |
| 1+ | 74 (15.3%) | 14 (11.7%) |  |
| 2+ | 81 (16.8%) | 22 (18.3%) |  |
| 3+ | 328 (67.9%) | 84 (70.0%) |  |
| IgM deposit, n(%) |  |  | 0.583 |
| - | 74 (15.3%) | 14 (11.7%) |  |
| + | 81 (16.8%) | 22 (18.3%) |  |
| C3 deposit, n(%) |  |  | 0.202 |
| - | 134 (27.7%) | 41 (34.2%) |  |
| + | 349 (72.3%) | 79 (65.8%) |  |
| IgG deposit, n(%) |  |  | 0.449 |
| - | 418 (86.5%) | 100 (83.3%) |  |
| + | 65 (13.5%) | 20 (16.7%) |  |
| C1q deposit, n(%) |  |  | 0.631 |
| - | 478 (99.0%) | 118 (98.3%) |  |
| + | 5 (1.0%) | 2 (1.7%) |  |

M: Median; Q1: 1st Quartile; Q3: 3st Quartile; MAP: mean arterial pressure; eGFR: estimate glomerular filtration rate; 24-UP: 24-hour urinary protein; URBC: urinary red blood cells; NLR: neutrophil to lymphocyte ratio; TBA: total bile acid; UREA: blood urea; SCr: serum creatinine; UA: serum uric acid; CysC: cystatin C; IgG: immunoglobulin G; IgA: immunoglobulin A; IgM: immunoglobulin M; IgE: immunoglobulin E; C3: complement 3; C4: complement 4; IgA/C3: IgA to C3 ratio; RASB: renin angiotensin system blockade; GC: glucocorticoid; ISKDC: International Study of Kidney Diseases;

**Supplementary Table 3. Detailed performance metrics for each fold**

|  | C-index | 60-month AUC | 84-month AUC |
| --- | --- | --- | --- |
| Fold 1 | 0.840 | 0.900 | 0.765 |
| Fold 2 | 0.621 | 0.618 | 0.772 |
| Fold 3 | 0.616 | 0.525 | 0.568 |
| Fold 4 | 0.681 | 0.717 | 0.731 |
| Fold 5 | 0.690 | 0.628 | 0.706 |
| Average | 0.689±0.091 | 0.678±0.142 | 0.708±0.083 |

**Supplementary Table 4. Sensitivity Analysis: Performance Comparison Between the Full Model and Model Without Serum Creatinine**

| Metric | Dataset | Full Model | Without Scr Model | Difference | Percentage Variation |
| --- | --- | --- | --- | --- | --- |
| C-index | Train | 0.730 (0.649-0.802) | 0.705 (0.633-0.778) | -0.025 | -3.40% |
| C-index | Test | 0.796 (0.684-0.899) | 0.756 (0.627-0.867) | -0.040 | -5.00% |
| 60-month AUC | Train | 0.735 (0.629-0.827) | 0.711 (0.616-0.804) | -0.024 | -3.20% |
| 60-month AUC | Test | 0.859 (0.750-0.956) | 0.795 (0.670-0.917) | -0.064 | -7.40% |
| 84-month AUC | Train | 0.756 (0.657-0.846) | 0.755 (0.658-0.837) | -0.001 | -0.10% |
| 84-month AUC | Test | 0.850 (0.692-0.973) | 0.771 (0.586-0.923) | -0.079 | -9.30% |

**Supplementary Table 5. SSchoenfeld individual test results for the proportional hazards assumption**

| Variable | chisq | df | P |
| --- | --- | --- | --- |
| gender | 0.308 | 1 | 0.579 |
| IgA deposit | 0.781 | 2 | 0.677 |
| ISKDC | 0.014 | 1 | 0.904 |
| SCr | 0.332 | 1 | 0.564 |
| T | 0.173 | 1 | 0.678 |
| GLOBAL | 1.590 | 6 | 0.953 |

**Supplementary Table 6. Number at risk and cumulative censoring rates at 60 and 84 months**

| Time point | Number at risk | Cumulative censoring rate (%) |
| --- | --- | --- |
| 60 months | 232 | 54.7% |
| 84 months | 92 | 75.5% |

**Supplementary Table 7. Performance comparison of baseline models and the final model on the test set**

| Model | C‑index (95% CI) | 60‑month AUC | 84‑month AUC | Brier score |
| --- | --- | --- | --- | --- |
| ISKDC‑only | 0.386 (0.238–0.512) | 0.572 | 0.558 | 0.108 |
| ISKDC + T | 0.389 (0.239–0.519) | 0.565 | 0.559 | 0.107 |
| Gender+SCr | 0.201 (0.074–0.337) | 0.589 | 0.561 | 0.099 |
| Final model | 0.796 (0.684–0.899) | 0.859 | 0.850 | 0.097 |

| Model | Dataset | Net Benefit at 10% threshold | |
| --- | --- | --- | --- |
|  |  | 60 months | 84 months |
| ISKDC‑only | Train | 0.015 | 0.105 |
| ISKDC+T | Train | 0.021 | 0.105 |
| Gender+SCr | Train | 0.018 | 0.109 |
| Final model | Train | 0.028 | 0.138 |
| ISKDC‑only | Test | 0.014 | 0.034 |
| ISKDC+T | Test | 0.013 | 0.034 |
| Gender+SCr | Test | 0.028 | 0.055 |
| Final model | Test | 0.047 | 0.061 |

**Supplementary Table 8. Net benefit at 10% threshold probability for baseline models and the final model**

**Supplementary Figure 1. Schoenfeld residual plots for each predictor in the final Cox model.**

**
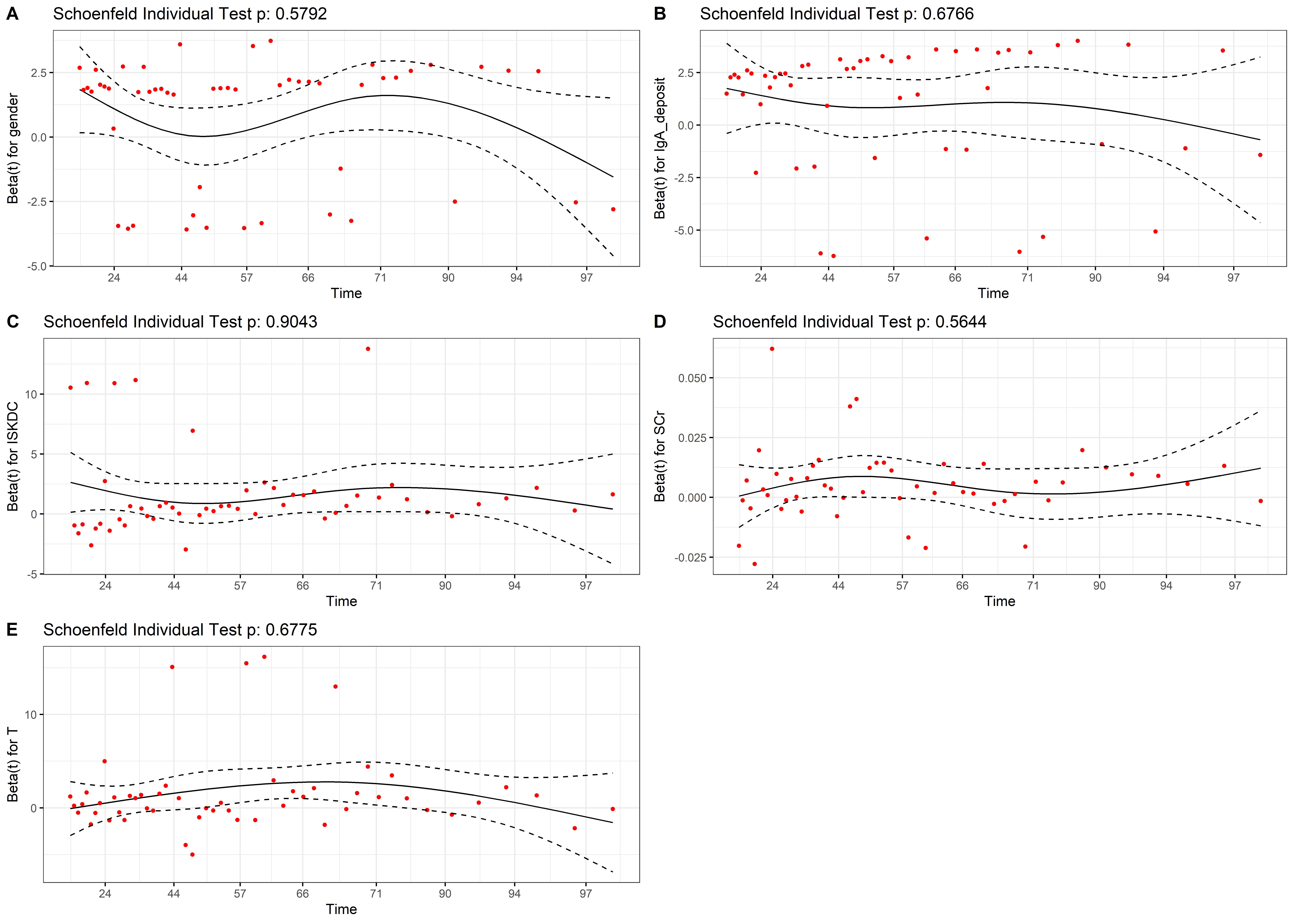
**

**Supplementary Figure 2. Spline fit of serum creatinine effect (restricted cubic spline with 3 knots).**

**
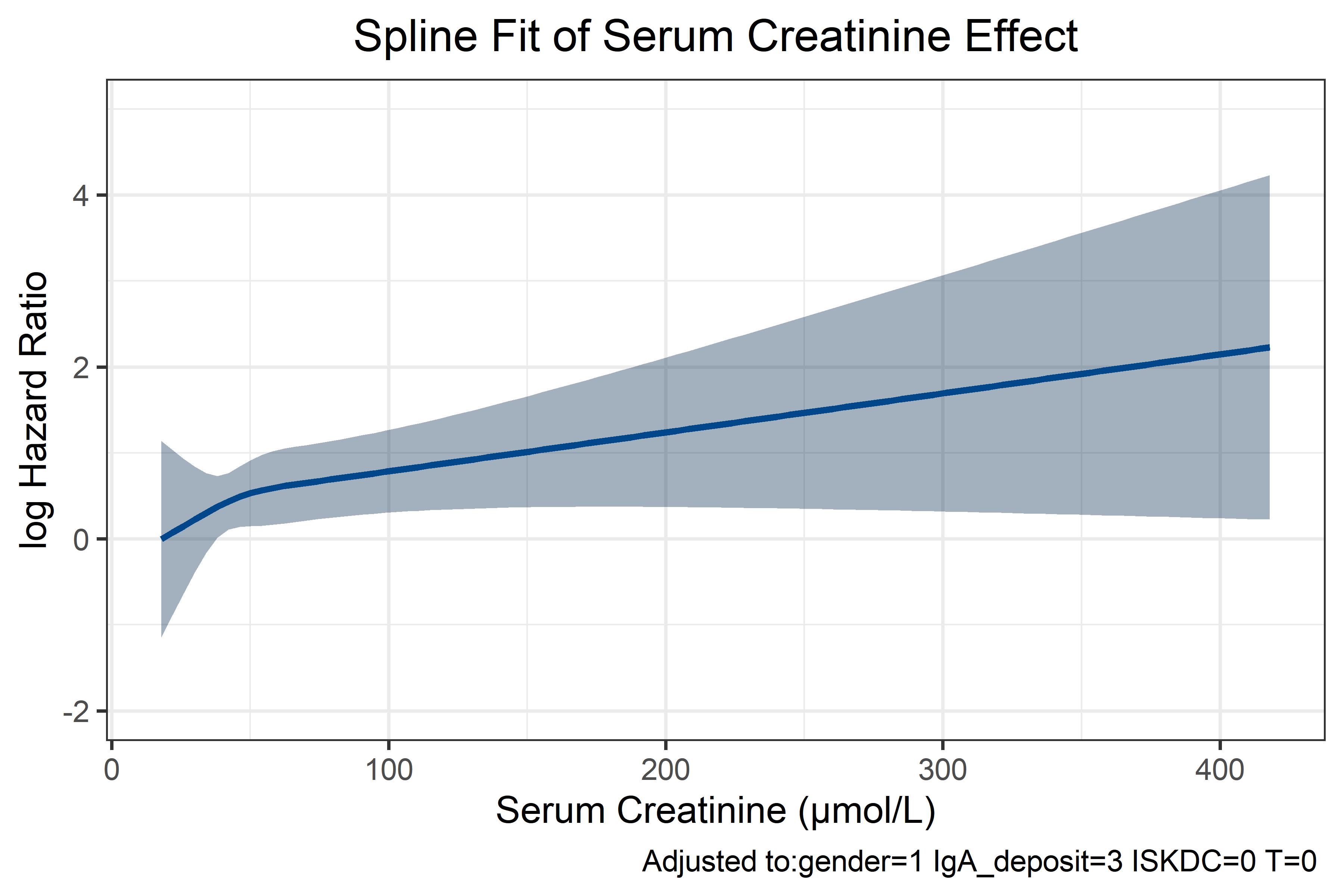
**

**Supplementary Figure 3. The 60-month / 84-month time-dependent ROC curves of the training set and the test set.**


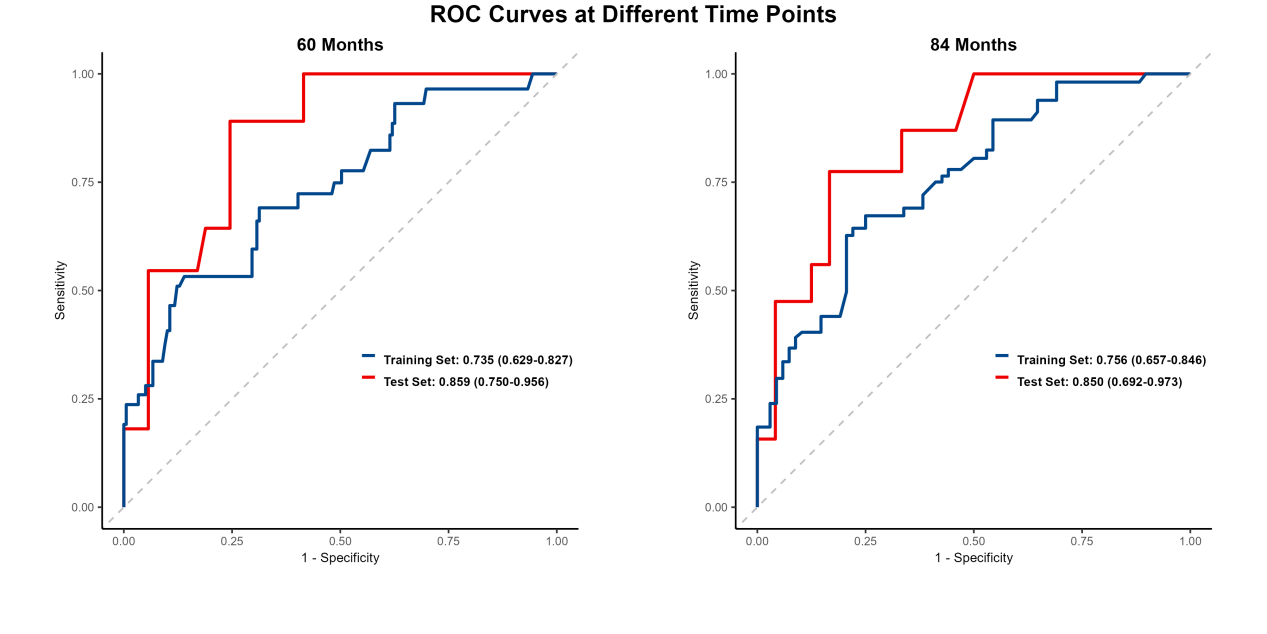


**Supplementary Figure 4. Decision curve analysis comparing the final model with baseline models at 60 and 84 months for training and test sets.**

**
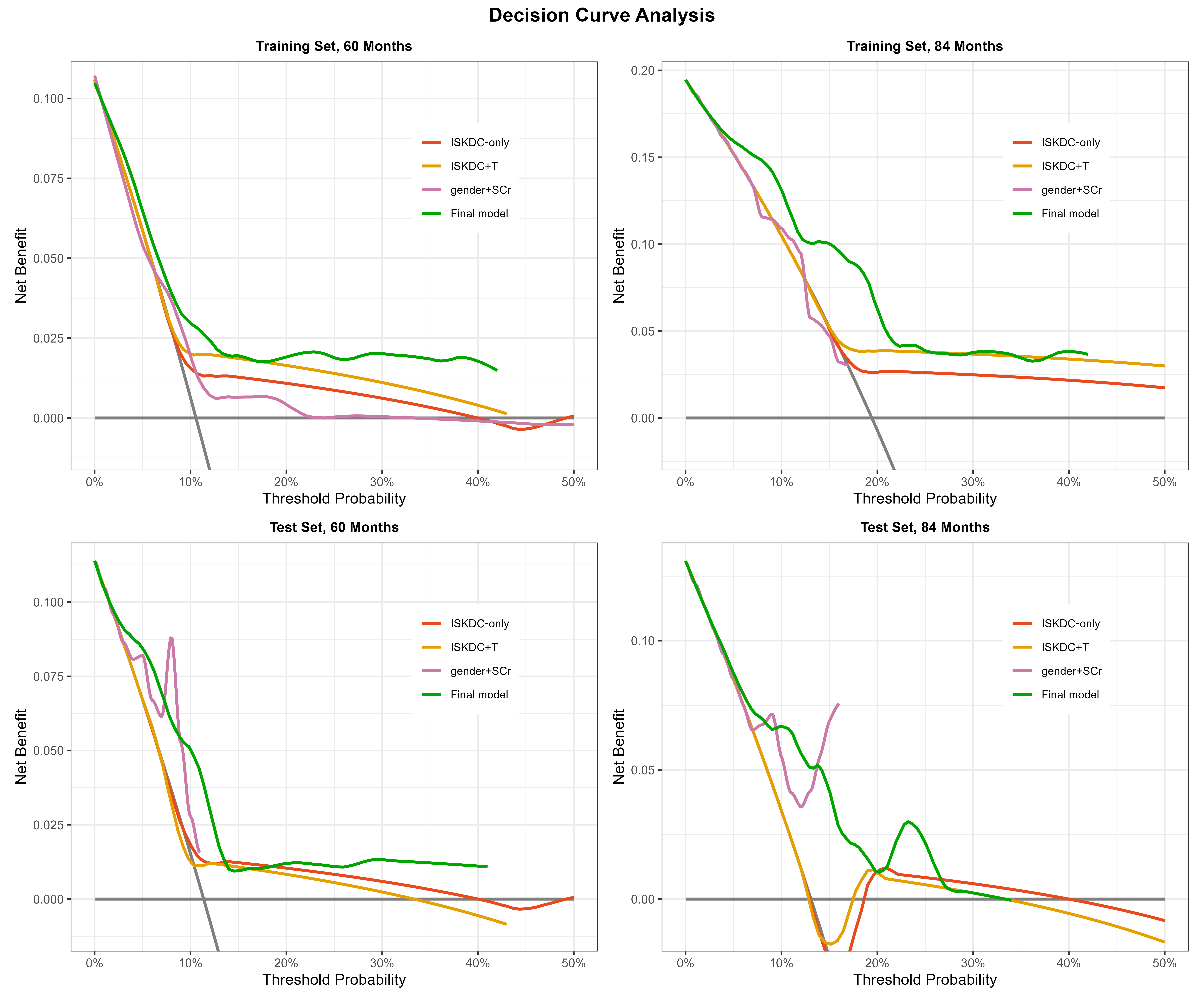
**

**Supplementary Figure 5. Kaplan-Meier analysis of event-free survival according to the five independent predictors in the final model.**


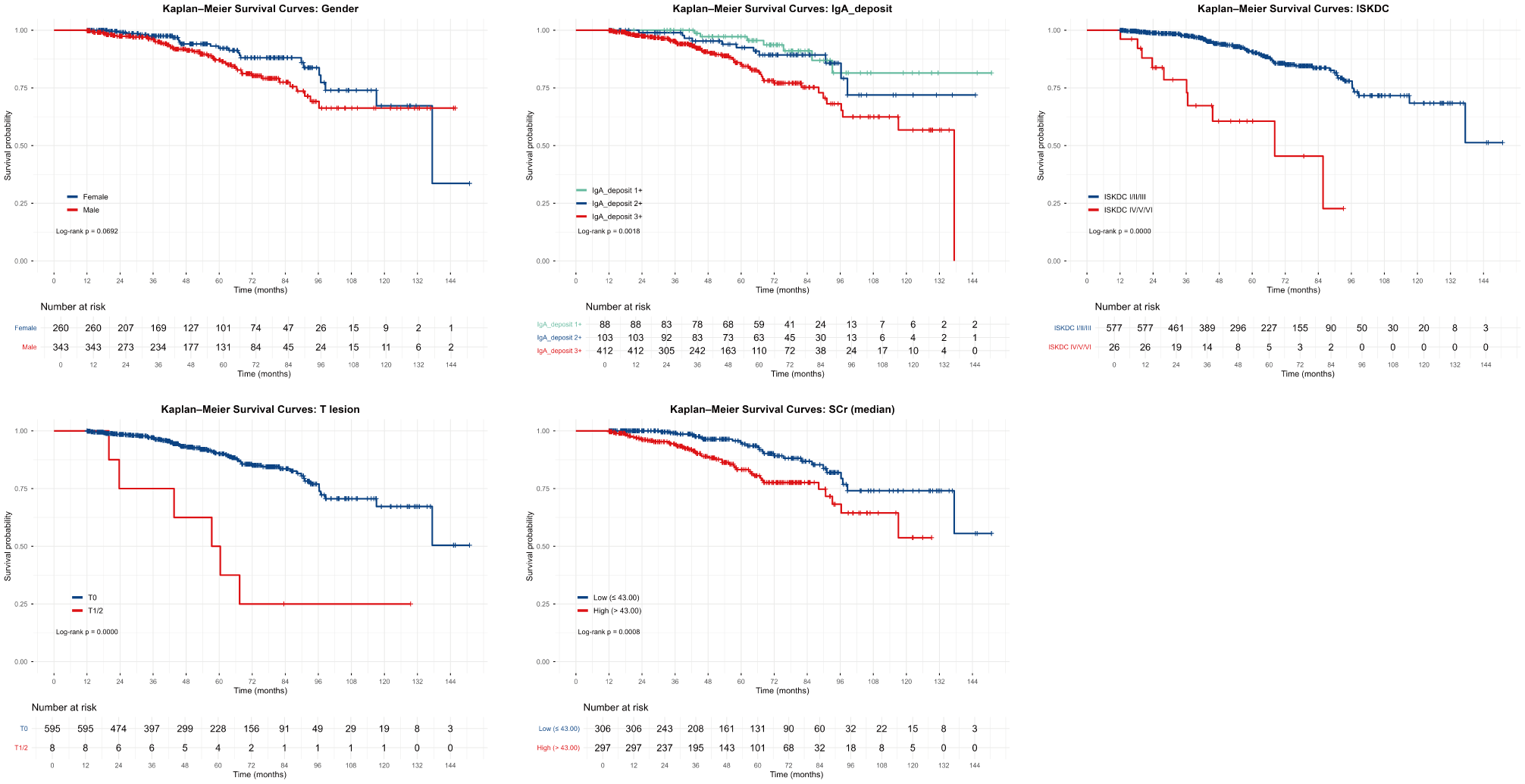

Supplement: Supplementary file 1 [file Supplementary_file_1.docx]
